# “It is a false safety net”: A qualitative exploration of multiprofessional staff experiences of insulin management in hospitalised older or frail adults with diabetes undergoing surgery

**DOI:** 10.1371/journal.pone.0332088

**Published:** 2025-10-07

**Authors:** Christina Lange Ferreira, Hellena Habte-Asres, Angus Forbes, Kirsty Winkley

**Affiliations:** 1 Care in Long Term Conditions, Faculty of Nursing, Midwifery and Palliative Care, King’s College London, London, United Kingdom; 2 Diabetes and Endocrinology, Hereford County Hospital, Wye Valley NHS Trust, Hereford, United Kingdom; Albert Einstein College of Medicine, UNITED STATES OF AMERICA

## Abstract

**Aim:**

To explore multiprofessional hospital staff’s experiences and perspectives of insulin use and safety review in older or frail adults with diabetes undergoing surgery.

**Method:**

Twenty-three semi-structured interviews amongst multiprofessional staff supporting four surgical wards (elective and emergency) at a single NHS Trust in a rural county in England. Interviews were recorded, transcribed and analysed using framework analysis approach.

**Results:**

Six main themes identified: (1) Transitioning through care: Misalignments across insulin related interacting components contribute to insulin errors (2) Coping with complexity, changes and ambiguity; at clinical, operational and organisational levels (3) Staff knowledge and confidence gaps affect insulin safety (4) Fostering patient empowerment; patient empowerment can be hindered by staff assumptions and inflexibility of hospital environment (5) Staff support systems; communication, power dynamics influence insulin safety (6) System learning as a cultural challenge; absence of systems approach and reactive to harm.

**Conclusion:**

This study provides valuable insights into reported complexities of managing insulin-related interacting components during surgical admission. Findings could inform how managers, leaders and organisations approach and consider multiple components implicated in safe hospital insulin use to build system resilience. Future research can build on insights from this study to develop interventions supporting system-based approaches to hospital insulin safety.

## 1. Introduction

An estimated 588.7 million people live with diabetes across the world, and this is estimated to increase to 852.5 million by 2050 [1]. As the prevalence of diabetes is rising, so is the number of hospital inpatients with diabetes [2]. In England, 1 in 14 people are estimated to live with diabetes; amongst hospital inpatients 1 in 6 beds is occupied by a person with diabetes; in some hospitals that figure reaches 1 in 4 [3,4]. Many of these patients have been admitted for reasons other than their diabetes and are therefore under the care of hospital staff who are not diabetes specialists. Internationally, there is recognised variability in the care people with diabetes receive in hospital [3,5,6]. A high proportion of people with diabetes in hospital are older adults [7] living with multiple long-term conditions and polypharmacy. Such patients can be even more vulnerable and may require extra assistance to manage their insulin therapy [8].

Insulin management in hospital is complex, due to the high number of interacting components at the patient, healthcare professional and context level. Insulin related errors in hospital happen frequently, with errors reported across high, low and middle income countries [9–13], at all stages of the medication use process (i.e., prescribing, transcription, dispensing, preparation, administration, monitoring and management) [10,14]. Insulin errors are associated with dysglycaemia, inpatient related harm such as hospital acquired diabetic ketoacidosis (DKA), hyperosmolar hyperglycaemic state, severe hypoglycaemia and even death [5,11]. Hyperglycaemia in the perioperative context has been associated with increased risk of post-operative infections, complications and mortality, and 28-day hospital readmission following surgery [15–18]. Insulin errors cause significant anxiety and distress to patients and can be associated with longer length of hospital stay [5].

Suboptimal diabetes care and insulin management in the UK perioperative context were highlighted by the Highs and Lows report from the National Confidential Enquiry into Patient Outcomes and Death [19]. Clinical guidelines to standardize the care of people with diabetes undergoing surgery have been developed [20,21]. Multi-component whole-perioperative pathway approaches and interventions which include multi-stakeholder working groups, staff education and patient empowerment have demonstrated effectiveness in improving the outcomes of patients undergoing surgery, with reductions to hospital length of stay, 30-day readmission, and post-operative complications [22].

However, despite high numbers of inpatients with diabetes in hospital, diabetes and insulin related knowledge and confidence amongst hospital healthcare professionals is often inadequate [5,23–25]. Equally, implementation gaps between recommendations, guidelines and actual care delivered are a persistent challenge [11,12,22,24]. The perioperative care pathway is particularly challenging, given the high number of transitions of care between teams and spaces in the hospital through to discharge.

Over the years, several initiatives and interventions to address hospital insulin safety have been implemented, with varying degrees of success [2]. Many of these have focused on prescribing elements of insulin use. Engaging stakeholders, developing the underpinning programme theory and considering contextual factors are recognized as essential to successful intervention development [26]. However, there has been a noticeable lack of theory development or involvement from key stakeholders in their development [27].

This study therefore aimed:

- To explore the experiences of National Health Service (NHS) staff in relation to insulin use and safety review in surgical settings, in the context of the older/frail adults with diabetes undergoing a surgical admission.
- To identify key factors associated with insulin safety and insulin use errors, from the perspective of NHS staff.

## 2. Methods

### 2.1. Design

This qualitative study is part of a wider co-design project to develop an intervention for Safe Hospital Insulin Use amongst older or frail adults undergoing a surgical admission to hospital (SHINE) [28]. This paper reports on semi-structured interviews with healthcare professionals as part of the initial phase of the SHINE Study. Reporting adheres to Standards for Reporting Qualitative Research (SRQR) [29] (S1 File).

### 2.2. Procedure

This study was conducted on four surgical and frailty wards (emergency and elective units) of a NHS general hospital. A consultant-led diabetes specialist inpatient team with diabetes specialist nurses was in place from Monday to Friday. The research site had not yet undergone a Diabetes Care Accreditation Programme (DCAP) but participates in the National Diabetes Inpatient Safety Audit (NDISA) [30]. Further details of the site are provided using The Joint British Diabetes Societies for Inpatient Care (JBDS-IP) [31] self-assessment checklist in S2 File.

### 2.3. Participants

Participant inclusion criteria were clinical and non-clinical staff involved in hospital care/transfer of care/insulin incident or safety review of older/frail adults with diabetes undergoing surgical admission for a minimum of 3 months so they had familiarity of local contextual factors. The study was promoted via recruitment posters at the hospital site; disseminated through official communication methods such as the Trust newsletter and email. The lead researcher (CLF, a female diabetes specialist nurse and qualitative researcher) attended meetings with clinical leads and the diabetes team to disseminate study information. Participants were provided with an information sheet and written informed consent was obtained prior to participation. A one off ‘thank-you’ £15 voucher was given to participants participating out of work hours.

#### Sample.

Purposive sampling was employed to recruit staff representing different professional groups and different roles in managing and reviewing insulin use in hospital. Inclusion of different ranges of age, roles, and professional experience were considered important to obtain a comprehensive perspective. Sample size was guided by Malterud’s Information power (IP) Model [32]. Given the study’s narrow aims and objectives, use of pre-established theory, and the quality of dialogue that could be established through in-depth qualitative interviews with an experienced researcher, it was considered 20–25 participants would be sufficient to achieve the study’s aims.

### 2.4. Data collection

Study recruitment took place between 12 April and 13 November 2024. CLF conducted 23 interviews aided by a piloted topic guide, sample questions can be found in S3 File. All 23 interviews were held via MS Teams according to participants’ preferences, lasted between 30–80 minutes and were audio-recorded with permission.

Consideration was given to trustworthiness criteria in qualitative research [33]. To ensure credibility, in depth interviews were used to generate rich data, and analytical debriefings with the research team occurred. For dependability, the analysis framework was underpinned by previously established guidance in using and applying framework analysis [34–36]. Regular discussion and reflection amongst the research team and use of a codebook provided a transparent process with a clear audit trail. With regards to confirmability, study findings are supported with anonymized extracts of participants’ contributions. As CLF was employed by the research site and thus worked in the same setting as participants, reflexive journals were kept throughout the study to document their thoughts, ideas, and personal interests. These were discussed amongst the wider research team (AF, HH-T and KW) who were all diabetes specialists and clinical academics without connections to the research site, in order to identify any potential personal biases.

### 2.5. Ethical considerations

NHS Health Research Authority ethical approval was obtained from East Midlands-Derby Research Ethics Committee (24/EM/0022) prior to study commencement. The job roles/titles of participants or specific site/specialty worked at have not been presented to minimise risk of individuals being identified.

### 2.6. Data analysis

Initial verbatim transcripts were checked against original audio recordings for accuracy by CLF and then uploaded to NVIVO 14 software to support data management. Ritchie and Spencer’s five step Framework Analysis Process [36] was used to analyse the data, as it allows for incorporation of prior knowledge and theory. CLF listened to all original audio recordings and read and re-read transcripts to familiarise themself with the data. CLF then undertook line by line coding of six interview transcripts, one from each of the different professional groups. This generated initial codes which were grouped into 13 categories. All researchers (CLF, KW, AF and HH-T) then met several times to review selections of transcripts, discuss codes and identify initial categories. An initial thematic framework with six categories and subcategories was then developed. Framework development incorporated a-priori knowledge and theory from Complex Systems thinking, Safety 2 and Resilient Healthcare principles [37] and a scoping review undertaken which identified and mapped hospital-related insulin errors and associated interacting components [13]. CLF applied the framework from a generated codebook to all the data transcripts. HHT, reviewed the analysis codebook and independently coded a proportion of the transcripts. KW and AF also reviewed a sample of transcripts during the analysis process. Regular research team meetings occurred to discuss differences in data interpretation and assess usability of the coding framework. As suggested by Gale et al [34] the ’other’ code was used for each category to ensure data that may not fit the framework was not missed. On completion of coding, CLF entered the data into framework matrices for each theme. Themes were then mapped and interpreted and became the results of the study. This process is illustrated in S4 File.

## 3. Results

### 3.1. Participant characteristics

Of the 23 participants, the majority (52%) were in nursing roles, 17% were doctors, and remaining staff were in pharmacy roles (9%), allied health professional roles (9%) and patient safety & governance roles (13%). There was representation from junior clinicians and clinical managers and leaders from each professional group. Three participants worked in the diabetes specialist team. Seventeen described their ethnic background as White, five as Asian and one as North African. Further participant characteristics can be found in Table 1.

**Table 1:** Participant characteristics.

No	Sex	Age (years)	Ethnicity	Years working in NHS	Professional group
P1	Female	30–34	White	7 years	Nursing
P2	Female	35–39	White	13 years	Nursing
P3	Female	50–54	White	19 years	Nursing
P4	Female	45–49	White	30 years	Safety & governance
P5	Female	55–59	White	33 years	Safety & governance
P6	Female	25–29	White	12 years	Nursing
P7	Female	55−54	White	10 years	Safety & governance
P8	Female	40–44	Asian	2 years	Medics
P9	Female	30–34	White	9.5 years	Pharmacy
P10	Female	45–49	White	14 years	Nursing
P11	Male	25–29	Asian	9 months	Medics
P12	Female	30–34	White	7 years	Allied Health Professional
P13	Female	25–29	White	11 years	Nursing
P14	Female	45–49	White	24 years	Nursing
P15	Male	40–44	North African	3 years	Medics
P16	Female	25–29	White	6 years	Nursing
P17	Female	30–34	White	12 years	Pharmacy
P18	Female	25–29	White	8 years	Nursing
P19	Male	60–64	Asian	20 years	Nursing
P20	Female	35–39	Asian	8 years	Nursing
P21	Female	45–49	White	21 years	Medics
P22	Female	60–64	White	22 years	Allied Health Professional
P23	Female	50–54	Asian	14 years	Nursing

### 3.2. Themes emerging from the data

**Fig 1:**
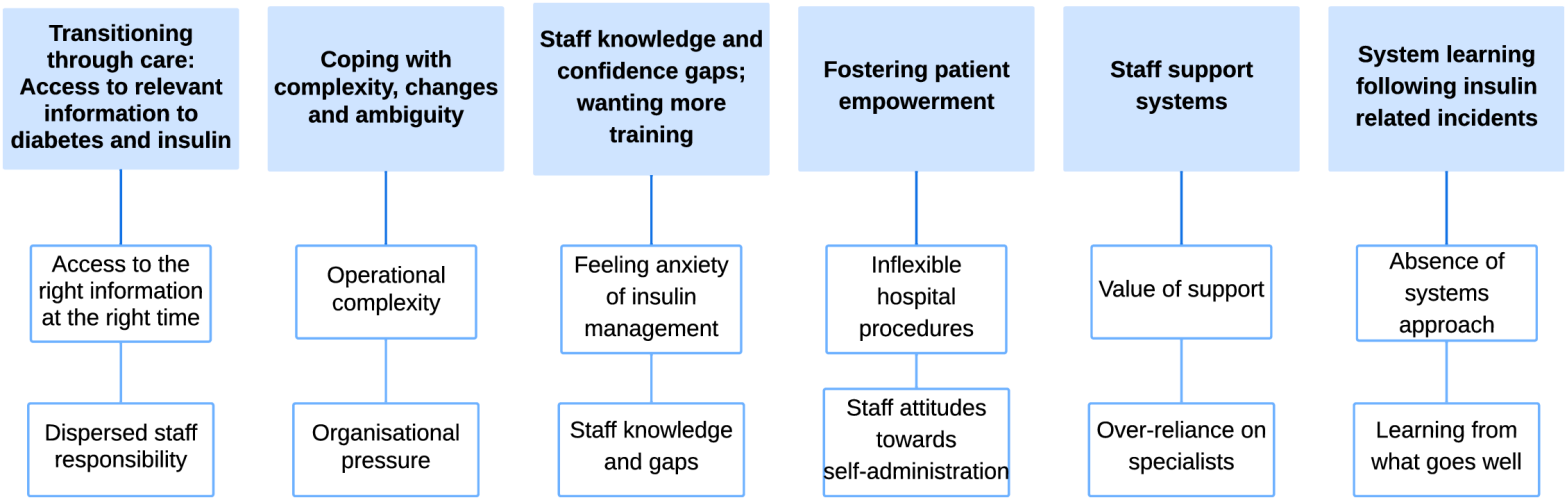
Themes and subthemes emerging from the data.

Findings are presented under six main themes (and sub-themes) which emerged from the data and are represented in Fig 1. Additional quotes can be found in Table 2.

**Table 2:** Summary of themes and subthemes & additional representative quotes.

Theme	Subtheme	Data excerpts (quote, participant identification number and professional group)
Theme 1: Transitioning through care: Access to relevant information to diabetes and insulin	**Access to the right information, at the right time**	• *“(...) one of the main challenges is trying to find out their units [of insulin] (...)that they take, especially if it is someone who’s quite frail and elderly and they haven’t got much support (...) if they don’t bring their medication in with them; (…) what devices are they using (...).” (P9; pharmacy technician)* • *It’s all right turning round and getting a fancy GP [medication] list (…). But (…), if the patient doesn’t take it (…), they don’t take it. So, patients always your first port of call.“(P17; pharmacist)* • *“the nursing team in pre op tell me it is not wonderfully easy (...) to know (…)whether they’re diabetic (...) before they see them. (...) if they’re a patient who are out of area, who don’t have access to EMIS or healthcare share (...) it all becomes (…) more tricky. (...),if patients are not the best historians and don’t come with the information, it can be possible that you don’t find them at all.”(P21, doctor)* • *“(…) especially if they’re trauma because they might have dementia or they (…) just don’t know.(...) It could take a whole morning [to find out their dose of insulin]. (...)invariably what will happen (…) patients, (...) either miss it or they get it much later in the in the day.” (P3; non-medical prescriber)* • *Patient comes into ED and they’re being clerked, (…), we’re trying to find the information with regards to how much insulin this patient is normally on (…) what happens with all this time spent calling one person, another person (…) this patient went into DKA unfortunately, before his surgery (…)“(P11, doctor)* • *“(…) if you are in ED seeing new patient (…) you usually go for the GP record (…) to see what medication is he on and (…) sometimes you will not find the exact dose but ‘as directed’. Usually the patient will know the dose, but sometimes you don’t know exactly what the dose is (…) especially if they are confused, and they come from home, they live alone and even if they have the insulin, we can’t get it prescribed because we don’t know the dose (…)” (P15, doctor)* • *“(…)But at the moment, I do think it’s slightly in different places [where all the guidelines are held]…” (P17, pharmacist)*
	**Dispersed staff responsibility**	• *“we’ve always had this relationship with surgeons, where surgeons aren’t taking ownership of it. The trouble is, we’ve seen so many people we can’t follow up everybody (…) to see whether they’ve stopped their diabetes medications or whether you know they’ve done this or that; (…), it’s a surgical thing, but the surgeons aren’t doing it(…) (P22; Allied Health Professional)* • *“You’re not sure if the patient’s actually been spoken to about their medication, I’ll come along, (…)mention it, and then they’ll be like, oh, what’s that? And then I’ve got to go through it with them, because no one’s actually explained to them that they’ve been started on it.” (P9; pharmacy)* • *“the only thing that we worry about is if people’s hearts and lungs are strong enough for anaesthetics. But historically we’ve ended up having to deal with medications and advice.(…) we’re supposed to then say, (…) this is this leaflet, take it away. This is what you’re supposed to do. (…) sometimes if they’re elderly (…) and they’re not very clear on it. And they’re not sure. And then we’re not sure, you know. (…) that’s why we say, (…) you need to speak to diabetic nurses; or the surgeons, because really it’s not an an anaesthetic issue. It’s a surgical issue, but we’ve landed with the role of trying to just, (…) trying to help and give advice” (P22, Allied Health Professional)* • *“Sometimes the EPMA will not be updated about the insulin (…) It is only in the take, maybe on the ward when the patient is reviewed by the consultant in the postake and then reviewed by the pharmacist and then you probably get the exact dose for the patient then. But in the theatre, on call and especially overnight, that is the problem” (P15, doctor)* • *“I think it might be in the too difficult box. (…) people, probably think it’s not their job to do. (…) there are specialist teams, (…) pharmacy, (…), perceived ownership is probably much broader, than some other incidents where it’s like right, well, this is within my gift, (…) my local area. I think that might be quite frightening for people, they maybe feel a bit disempowered.” (P14; nurse)*
Theme 2: Coping with complexity, changes and ambiguity	**Operational complexity**	• *“in terms of our patients (…) who are pre op and awaiting theatre. They’ll be nil by mouth and often the communication from theatres, if they’ve been cancelled; they don’t tell us in good time and I think that can lead to them missing meals...” (P13; nurse)* • *“Sometimes [difficult to administer insulin on time], (…) because of the pressures and how busy it is. (...) you can ask what time they’re going to theatre, but a lot of the time they can’t give you an exact time, or sometimes they don’t even know if it’s going to be morning or afternoon.(…).” (P16; nurse)* • *“If we do a drug round at 6, but then the evening meal comes at 5:30. (…) we have to give their insulin at 5:30. (…) then, it’s too early (…) to give the rest of the medication or like we’re in the middle of admitting someone or settling someone who’s just come from surgery, (…)you have three patients with insulin all at the same time. (…) OK, who do I go to first?”(P20; nurse)* • *“I’ve had one yesterday, (…) the insulin wasn‘t available; it was supplied and then wasn’t given and the patient,(…) went into a DKA and ketones (…) were really high.” (P17; pharmacist)* • *“I think there has been a couple of patients where we forgot to let diabetes know that we were starting [NG feeds] and (...) they’re hypo because we’ve changed the regimen and given them a break at like the wrong times for their insulin(...)” (P12; Allied Health Professional)* • *(…) we’ve had patients that come into ED, it’s not been clerked and patients have missed insulin for like 6–8 hours and they’ve not said anything (…) even well patients that could. I think it’s a... It’s a false safety net. They’ve come into hospital and they think they’ll be OK. They’ll be looked after. But actually if we’re not aware (…) They won’t be safe.” (P7; safety & governance)*• *“(…) if we can’t find the actual dose, you know, it’s like, oh, what do I do? (…) what do I give [prescribe] them; if I give them some something and It’s not enough.... If I give them too much...” (P3, nurse & non-medical prescriber)”* • *“[insulin] just seems to be something that people don’t bring in with them [to hospital]. (…) Oh, well, you’re a hospital. You’ve got insulin here. (…) See a bit of a running theme, especially with older people. (…)” (P 18, nurse)*
	**Organisational pressure**	• *“I do worry that maybe they rush, instead of actually taking their time.(...) that’s (...) quite evident when clerking patients. (...) A lot of insulin (...) gets missed off clerking. (...) they’re just so busy going from patient to patient, ward to ward” (P07, safety & governance role)* • *“(…)when you’re down in ED clerking patients,(…)there’s a pressure of how many patients you have to see within the set timeframe.(…) So how much time you can spend on per patient? “ (P11, doctor)* • *“Certainly within the climate at the moment we’re boarding four or five extra patients all (...) the time. We haven’t increased staff, we’ve got a high agency reliance...” (p14; nurse)* • *“(...) I would like to check insulin with the patient at the same time (…) is this what you normally take (...) But it doesn’t always happen.(...)Especially with the acuity that we have. When you’ve got four people sleeping in the corridor and six people to get in through A&E, (...) I think it could be more reinforced that we actually check at the bedside with the patient rather than just in, in the corridor going. Oh, can you check my insulin?” (P18; nurse)* • “*Staff change, we get new people. People leave.(...)The insulins change. (…) a constant cycle of going back and forth between all of them.” (P13; nurse)* • *“[the rotations of junior doctors] has a lot of impact because whenever you are in a new environment, you are under more stress, so you are more; you can make more mistakes.” (P8; doctor)*
Theme 3: Staff knowledge and confidence gaps; Wanting more training	**Feeling anxiety of insulin management**	• *“There’s a lot that I think I don’t know. (…) I feel like... Diabetes could be (…) watered down to like: Oh, it’s just a blood sugar that that goes all over the place. And you give them tablet. You give them insulin. That’s fine. It’s gonna work. But actually, it’s it’s more than that (…) it can be challenging.” (P20; nurse)* • *“I think [IV insulin], that freaks everyone out, to be honest. I’m not a fan of that.” (P16; nurse)* • *“(…) Diabetes as the topic is a minefield, isn’t it? It’s so massive.(...) It scares me. It’s really overwhelming (…) it seemed like years ago there were only a few insulins, and now there’s so many different treatments, it’s hard to keep up with (...) (P2; nurse)* • *“insulin is one of those medications (…) we have to be much more careful with (…) we have heard on the news (…) giving the wrong dose does kill (…)people have died, whether intentionally or accidentally. (…) I do [resonate with healthcare professionals feeling scared of insulin] I absolutely do” (P11, doctor)*• *“So because I am afraid from the hypo rather than the high, especially for the elderly people, so especially for night be very (…) cautious (…). Especially those almost not eating, very confused, (…)” (P15; doctor)*
	**Staff knowledge and gaps**	• *“it is harder because medications are changing so often now and the brand names are changing all the time. So, keeping up with what is what, and what means what, I do find difficult, when you’re not just doing diabetes, I find that hard.” (P12; Allied Health Professional)* • *“I know it sounds awful, but until they have made an error and then they have had that reflection and learning on it and familiarise themselves with policy procedure, it’s not routine...” (P13; nurse)”* • *“Some people have difficulties with regards to (…) the prescription part (...) there is actually order sets available on the on the EPMA. (…), I guess some people don’t know that (...).” (P11; doctor)* • *“(…) I’ve seen a few incidences where the doctors have said, right, yeah, they can come off the sliding scale now and the nurse take that for literal, I’m going to stop the sliding scale right now. And they don’t check things like, have they had their usual insulin or have they eaten? So they don’t technically follow the correct procedure in the safe discontinuation (...).”(P13; nurse)* • *“(…) if they come in at a weekend (…) seen by the ward team, (…) often straight away, put onto a variable rate infusion, they stop everything else and (…) starve them for long periods.” (P21; doctor)* • *“in this case (...) they didn’t know that someone with type 1 diabetes needed to have their basal insulin and be put on variable rate if they’re nil by mouth for several days (...) that foundation understanding wasn’t there for these healthcare professionals” (P1; nurse)* • *“(...) the fluids seem to be the hardest component of people getting right, and the monitoring (...) making sure (...) we’re running the right fluid at the right time at the right rates.” (P17; pharmacist)* • *“We just have the safe use for insulin e-learning (…) because it’s such a big issue, and there’s lots of incidents around it, (…) more mandatory training would definitely benefit the staff.” (P2; nurse)* • *“they’ve trialled clinical practice weeks where diabetic nurse pops to the ward and try and grab the staff that are free. (,) you will bet that everything will go wrong (...)So it’s just you need that protected time off of the ward. You need it factored into the rota.” (P13; nurse)* • *“you see the student nurses come in (…) everybody injects differently (…) in a different place (…) nobody (…) teaches them about, you know, using the clock technique or using different sites and things like that.(…) They don’t get it, you know? They’re just like, oh, yeah, insulin. It’s just another injection. Oh, I can do that. (…) We need education, definitely. And (…), empowering the patients to take control..” (P18, nurse)*
Theme 4: Fostering patient empowerment	**Inflexible hospital procedures**	• *“I think (…) the whole hospital inpatient system anyway, is just it’s not set up to be flexible. (…) you get up at this time, you have your meals at this time. There’s no inbuilt flexibility within that at all is there?” (P14; nurse)* • “*a patient will be like, say, oh, what’s my blood sugar? (...), oh, I’m going to have this much of food (...) I think I’m going to give myself 14. And you’re like, but my EPMA only says 10 units. Like, what am I going to do?” (P20; nurse).* • *“the prompt has gone from EPMA to self administer.It sort of like, reduced the amount of people who are actually self administering.” (P18; nurse)* • **“***So if I’ve looked through the notes, then I’ve seen that they’re usually independent with their management. (…) I say to them, do you need any help? Are they letting you do what you need to do? (...) And I would say 90% of the time they would reply to me, no, they’ve locked my insulin away. I can’t do anything myself. Which is really sad.” (P6, nurse)* • *“I have heard from patients that they were dissatisfied because they weren’t able to self-manage in the way (…) they should have been able to. And I don’t think that’s always reported.” (P14; nurse)*• *“we’ve got so much red tape around self administration, we make it so difficult for ourselves. We just almost take everybody’s autonomy (…) as soon as they become an inpatient.” (P17, pharmacist)*
	**Staff attitudes towards self-administration**	• *especially with the elderly, (…) maybe they think that they’re not going to understand. So they don’t [involve them in decision making]. (P9, pharmacy technician)* • *“I think when you’ve got to think of the reason why patients come into hospital in the first place. So if they’ve come into hospital and they are obviously frail, they’re elderly and they’ve come in because they’ve had an accident, they’ve fallen, they’ve broken their hip... Incidents of postoperative delirium are very high. You wouldn’t want them to have their control of their insulin if they had a delirium because they could potentially do themselves... (…) some mischief.” (P3; non-medical prescriber)* • *“Honestly, I think if we actually allowed [patient’s] to self administer [insulin], I think that would (…) save us from missing it (…) with their meal time, especially (…).” (P20; nurse)* • *“[when self-administering insulin] everything runs so much more smoothly and they just have such a better patient experience (…).****”*** *(P6; nurse)* • *“Well, I suppose it’s that knowledge we’ve always been taught to lock all the medications away, because that’s the policy. Is it in the policy that insulin can be left out? (...)Oh, it is, is it? Oh, I never knew that.” (P16; nurse)* • *“Well, talking about hierarchy, I mean we can put the patients at the bottom of our hierarchy, can’t we because they’re, you know, what do they know about their own health? Goodness me, they’re not professional healthcare providers, are they? I think there’s still a little bit of that, that kind of attitude. (...)listening to the patient’s voices. It’s difficult for a lot of people. They don’t, (...) view the patients as experts in their own bodies or their own health conditions, (…) that’s challenging. (...) (P14; nurse)* • *“I think that all that we know best is really amplified particularly with with older and frailer patients. And if you add in people who are in receipt of formal care, or people who may have, you know, cognitive impairment (…) there’s another layer added on again.” (P14; nurse)* • *“it was an internal struggle. My brain was like, am I going to do this? Am I just going to follow the patient when I’m so used to, like, get a prescription telling me what to do? (…) it helped discussing it with my colleagues(…)there’s different backgrounds(…) of nurses isn’t it.I’ve worked with nurses who trained in the UK and (...)other countries.(…)talking about that experience and (…) realising that I’m not alone in that journey (…)t made me feel that actually, you know what, it’s fine.” (P20; nurse)* • *“No, its the nurses, I am not involved in self-management.” (P15, doctor)*
Theme 5: Staff support systems	**Value of support**	• *“(...) we’ve got a really close-knit team and they’re quite a new team, (...) They’ve relied on each other heavily. I know they can approach each other and same with our team of doctors.”(P13; nurse)*• *“I have to admit that the diabetes team in in the hospital has been really helpful, if we have like questions about doing certain things (...) Pharmacy as well has been really helpful about like discussing the different types of insulin we have.” (P20; nurse)*• *“(…) I tried to get familiar with the Intranet guidelines and prescribe it then according to the (…)guidelines and you can never go wrong with those.” (P11; doctor)*• *“(…) the most important thing is that you have to give the confidence to the junior doctors, that they can ask questions.” (P8; doctor)* • *“So if its a busy day (...) consultants, the senior team would be busy with their jobs and, (…) they might be a bit more… (…). Pressurized, a bit stressed with carrying out their own work and, you know us, people asking them simple questions might not necessarily be the best time, best situation. It’s just like additional workload for them, I guess for for something they might think ohh it’s something very simple. Why did you even ask? (…) So yeah, sometimes it does feel that way.” (P11; doctor)* • *“there are some situations when the patient is very unwell and would (…) definitely require senior inputs (…) for example, diabetic ketoacidosis (…) a medical emergency that you (…) definitely want to escalate to a seniors before you do anything. And I guess senior availability for help?” (P11, doctor)* • *“some medical registrars are very good. So when the surgical FY1 or SHO would call them and ask them, they would be very happy to help them depending on what kind of workload they are having. And some are not very supportive. So, I think that also makes difference.” (P8, doctor)* • *And you can imagine how many surgeons we’ve got here, and they don’t like to be told. So what can we do, you know?“(P22, Allied Health Professional)*• *“It is easier following verbal handover. (...) Especially for someone you are (...) concerned about (…) we should give verbal handover in addition to writing in the notes.” (P15; doctor)* • *“(...) because of the culture of protected mealtimes, I don’t think they [nursing staff] feel like they’re able to [administer insulin at mealtimes]. (...) Because I’ve seen, (...) people reprimanded for trying to administer drugs around mealtimes. Where the nurse in charge has said to them things like, you should be helping with meals.” (P6; nurse)*
	**Over-reliance on specialists**	• *“If blood sugars are high and no one seems to be doing anything about it, (…) just checking that they’ve had a diabetes referral.” (P12, AHP)* • *“Automatically teams out there think oh contact them [diabetes specialist team], and they can do it. (…) sometimes is a good thing, that they are so there, but also they are almost relied upon to to come and do it and come and sort it out as well.” (P4, safety and governance)* • *“I think a lot of our junior doctors are dependent on the diabetic nurses(...) they’ll say like in working hours: Oh, let’s wait for the diabetic team to come and review or the nursing staff can ring the diabetic team and get an immediate response. Over the weekend.... At times it can just be a bit of a free fall. And they don’t often make changes to patients regimens. (…) They could just be having doses of that actrapid and doses of actrapid and doses of that actrapid(…).” (P13; nurse)*
Theme 6: System learning following insulin related incidents	**Absence of systems approach**	• *“My feeling is that yes they are reported but a lot of the time it’s not looked into why it happened.(...) sometimes, when it is reported, it’s almost you can be used as a bit of a stick to beat people with (…) it should be an opportunity really.” (P4; safety & governance)* • *“(…)Therefore you must, you know, reflect on it and tell us all about, you know, the mistake that you’ve made. (…) I’ve seen some of the reflections. (...) They turn into a timeline of events. (…) they don’t explore the the background; people are reluctant to put in writing what the, (…) what those contributory factors are, it’s not really explored in any depth. So I don’t think we manage any of our medication incidents in a way that truly reflects learning.” (P14; nurse)* • *“I’m not a diabetic expert by any means. [a tool to support review of insulin incidents would be helpful] Absolutely. (…) I find it quite overwhelming. (…) one of the diabetic nurse specialists has reported an inphase before (…) I didn’t fully understand. The ins and outs of everything. So I went and spoke to that CNS to say(…) Can you explain a little bit to me what should have happened?” (P2, nurse)* • *“It’s whether they understand it’s an incident (...) it is the (...) diabetes team that report them more so than the ward staff (...) People almost (...) don’t recognise them as an incident sometimes...” (P5; safety & governance)* • *“if there’s an error that’s been made or an issue that’s happened, (...) and the patient is doing OK (...) It still, (…) has a much lower emphasis placed on it than if something fairly catastrophic has happened, in which case you know everybody jumped in (…) really rapidly.” (P14; nurse)* • *“ we had the LFPSE questions put in to the system, their national, we can’t get away from them. People don’t like them, don’t understand the questions. It’d be good if they disappear, but we know they won’t disappear because it’s a national requirement.” (P5; safety & governance)* • *“ to do an inphase, for example, that takes a good 10-15 minutes. Does anyone want to stay after their shift, not to get paid, for an extra 15 minutes? No.”; “You just don’t have the time to report, (...) that is probably the biggest factor why things don’t get reported in general.” (P16; nurse)* • *“its just looking for er the person that’s done it (…) It’s that ethos still in the NHS, (…) that you want something, (…) tangible to... when actually it’s probably like a perfect storm with everything.”(P12;Allied Health Professionals)* • *“I think people are worried about the reaction from the patient or the relative. They’re obviously worried about, maybe anger towards them, (...). People get worried about that or they get worried about, are they going to get the blame for it? (...)” (P5; safety & governance)* • *“Usually... I think people are sort of... won’t always want to fess up. (...) there’s a lot of people who will be like, oh, my God! Oh my God! (...)I think that... I was taught how to lose my pin in university in my last year of university and it was all ways you’re gonna lose your pin and all the ways you can get kicked off the register. And I think that (…) put in the fear of everything into you.(…).” (P18; nurse)* • *“I think because you know with the inphase I think, the the people who are working in that ward, (...) they would know which person did this? So then they would know, and then to some extent. Then that person would feel targeted(...)” (P8; doctor)* • *“But it also could be a cultural thing (…). It’s sort of a sign of, you know, you’re not actually doing very well.(…)I think it’s over will they think less of me as a nurse. Or would they think less of me as a colleague? (…) oh, I’m new. So, obviously I was the one who’s going to make the error.” (P18; nurse)* • “So the issue is not that, yeah, so they [doctors] don’t understand that basically incident being reported, is not against them...” (P15, doctor)
	**Learning from what goes well**	• *“Most of time we learn from our mistakes. (…) I think we don’t appreciate people who do well (…)” (P8, doctor)* • *“There are pockets where things work well and that generally is, you can see really engaged and present leadership in those areas. I think if we’ve got that right, if (…) people are able to get out from behind the computer and go in, you know, sort of show staff by doing and by supporting them, erm, and patients, of course, you know. That really makes the difference “(P14, nurse)* • *“I’m not always convinced [that learning from incidents is shared] for any incidents, not just insulin. I mean, it will be captured in the ward huddle, (…), it’s who’s available to attend.(...)And how that is shared with staff who aren’t available to attend?” (P7; safety & governance)*

#### Theme 1: Transitioning through care: access to information relevant to diabetes & insulin.

Safe insulin use in hospital was presented by participants as an interconnected dynamic and adaptive challenge across the patient journey from pre-op to admission, theatre, recovery, ITU, ward care and discharge.

**1.1. Sub-theme:** **Access to the right information at the right time:** Participants described the importance of having up-to-date status information on the patient at all stages of treatment. Failure to handover the right information as participants transitioned through services was thought to be a major cause of insulin errors:

*“And (...) I can fully say that, yeah, I haven’t given insulin on time because of not realising the patient is on insulin or not having it handed over; You know, drama on the ward; and you know the high high acuity, you know, sometimes other things will take priority.” (P18; nurse)*

Staff spoke about problems accessing certain IT systems, finding the right guidance on the intranet, and a general lack of record integration across departments. The quality of clerking on admission was also variable. Staff reported being uncomfortable relying on often out of date General Practitioner reports which might not have correct dosage information, or on frail patients for accurate information when they were not always the best *“historians” (P21, doctor)*. Patient factors such as clinical status, communication or cognitive impairment, which may be more prevalent in older adults or those with frailty posed additional challenges to staff obtaining accurate information for managing insulin safely. Inaccurate or missing information resulted in patients missing or getting their insulin much later in the day.

**1.2. Sub-theme:** **Dispersed staff responsibility:** Multiple tasks being undertaken by different professionals across the care pathway led to no one being fully responsible for overseeing insulin management:

*“The trouble is, we’ve seen so many people we can’t follow up everybody (…) to see whether they’ve stopped their diabetes medications or whether you know they’ve done this or that; because it again, it’s a surgical thing, but the surgeons aren’t doing it (…) (P22; allied health professional)*

Against a backdrop of operational pressures, staff were sometimes confused as to who had spoken to the patient when they were put on new medications or different insulin regimens in hospital. Patients needed to be educated about these changes for their own safety, but it was not always clear who had responsibility for doing this.

#### Theme 2: Coping with complexity, changes and ambiguity.

Participants described how working within a dynamic environment with constant change, unpredictability, and ambiguity impacts on insulin safety.

**2.1. Sub-theme:** **Operational complexity:** Having to align patients’ insulin doses, their mealtimes and their surgery was particularly complex. When patients were transitioning between theatre and the ward, it was difficult to ensure timely coordination of insulin and meals. In some cases, patients from cancelled operations still ended up missing their meals. In addition, ward routines such as drugs round timings did not always align with insulin administration times:

*“So on the drug round, (…) Say you are there at 8:00 o’clock and you had an insulin at 7 (...) hopefully, the night staff have given it at 7. But what I’m trying to say is sometimes until you go into that drug round, you might have missed one. If it falls out of the normal drug round.” (P13; nurse)*

Expectations by patients that the hospital would just take care of them, meant many older adults commonly did not bring their own insulin devices with them, or brought insulin that was out of date. However, accessing the right insulin was often difficult, particularly out of hours, which sometimes caused critical delays.

**2.2. Sub-theme:** **Organisational pressure:** The socio-economic backdrop impacting NHS services was evident as staff discussed how they were “*always in a time of crisis” (P14; nurse).* It was clear that patient flow and high workloads could lead to clerking mistakes, which could introduce insulin errors from admission to discharge:

*“I do worry that maybe they rush, instead of actually taking their time. (...) And I think that’s (...) quite evident when clerking patients. (...) A lot of insulin (...) gets missed off clerking. (...) they’re just so busy going from patient to patient, ward to ward” (P7, safety & governance)*

Pressures associated with moving high numbers of patients through the hospital system, with patients on trolleys in corridors whilst awaiting a ward bed, made insulin management challenging. Patient flow and staffing pressures could also lead to delayed full medicines reconciliation or adaptations in discharge procedures which meant discrepancies were not identified in a timely manner. Rotating junior doctors, the main insulin prescribers, constantly faced stress and uncertainty of prescribing in a new environment. In addition, high staff turnover and a reliance on agency staff, also complicated attempts to standardize procedures or deliver local education on insulin care and safety.

#### Theme 3: Staff knowledge and confidence gaps; Wanting more training.

Professionals felt empowered when they had up to date knowledge, skills and confidence, but identified common knowledge gaps and fears regarding insulin management in hospital.

**3.1. Sub-theme:** **Feeling anxiety about insulin management:** Many participants in the interviews described diabetes management and insulin as challenging, and even *“overwhelming”* (*P2; nurse*). Some reported fear of causing hypoglycaemia, especially in the older population, which could lead to reluctance to adjust insulin in the context of hyperglycaemia. Some staff lacked confidence with newer diabetes technologies; leading to a reluctance to adjust and proactively manage insulin:

*“I do think there’s a culture around insulin. Oh I can’t. I can’t adjust it. You know, I’m not allowed to. (...) And I know a lot of people are this, you know, insulin and warfarin, I think are the two drugs that a lot of people have a lot of anxiety about.” (P3; non-medical prescriber)*

**3.2. Sub-theme: Staff knowledge and gaps:** Healthcare professionals described how difficult it was for them to remain up to date with clinical changes and developments, particularly “*when you’re not doing just diabetes*” *(P12; AHP).* Specific knowledge gaps included monitoring and fluid management whilst on Intravenous insulin (IV); transitioning safely from IV to Subcutaneous insulin, and using insulin pumps:

*“I think there’s a definite (...) educational gap for a lot of people around what (...) [insulin pumps] can do, what they can’t do… because if you don’t really know, then you get a bit suspicious and frightened of them, don’t you? So you avoid them and you do whatever you used to do before.” (P21, doctor)*

Participants remarked at the lack of focus on diabetes and insulin management during their undergraduate training. Staff reflected that learning was often ad hoc, arising from mistakes in clinical practice. Most staff wanted more mandatory hospital training but also protected learning time to capitalize on this training. For many though, training was not thought to be an organisational priority:

*“The education is always the first thing to be cancelled. (...) it will always have a lower priority than operational issues. Which (...) in times of crisis is it is understandable, but when we’re always in a time of crisis (...).” (P14, nurse)*

#### Theme 4: Fostering Patient Empowerment.

Staff recognised the value of empowering patients to self-administer their insulin in hospital for better patient experiences, although implementing this could be challenging.

**4.1. Subtheme:** **Inflexible hospital procedures:** Whilst the hospital has a self-management policy for insulin therapy, there were several service barriers hindering patient empowerment. A system update to the EPMA system had made it harder to prompt and record self-administration. Hospital policies and cultures such as locking away their medication on admission, and having to adhere to prescribed meals and mealtimes took away patient control:

*“As soon as they come through the door, we whip everything away from them. We lock it all in a cupboard.”; “I think... We do take a lot of their autonomy away, patients’, when they come into hospital, not just with insulin, but with everything.” (P3; non-medical prescriber)”*

In addition, allowing self-administration of insulin could lead to situations of conflict where patients’ prior knowledge and their new regimen in hospital did not match, and there was little guidance for staff on how to navigate the situation effectively.

**4.2. Sub-theme:** **Staff attitudes towards self-administration:** A hierarchical culture within the hospital which failed to recognise patients as experts in their own insulin management restricted their opportunities further. Assumptions were made about whether older patients could manage their own insulin without even checking with them:

*“But even patients who come in, I think they’re assumed, oh, they’re frail, I’ll do their insulin. They couldn’t possibly do their insulin. And I think we massively underutilize the self-administration policy, we don’t assess enough patients.” (P13; nurse)*

Fluctuations in a patient’s clinical and cognitive status required constant monitoring, and whilst it was sometimes safer for staff to administer insulin at points, the process of giving back control when patients became able again was not adequately embedded into practice.

*“They could be completely fine at managing their insulin at home. They come into hospital, have surgery, they become delirious and then the nursing staff take over with their insulin. And then (…), even when the patient has regained that capacity, then we aren’t very good at going. Oh, OK, right. You can self-administer your insulin (...)” (P13; nurse)*

#### Theme 5: Staff support systems.

Accessing support, advice and encouragement on diabetes and insulin management from colleagues and specialists was clearly valued by the staff and assisted with effective treatment.

**5.1. Sub-theme:** **Value of support:** Participants valued having support from a range of colleagues including the specialist diabetes team and pharmacy. Camaraderie within teams helped facilitate better communication and knowledge transfer. Participants spoke of the importance of creating safety cultures where staff feel it is acceptable to ask questions. However, professional hierarchies sometimes had a negative impact on insulin safety. For example, previous negative experiences when checking with colleagues could affect staff confidence to challenge in the future:

*“Because the clinician should say, you’re right to check, let’s just have a quick look. Sadly, they don’t. They don’t always get that response (…), it can be a very negative response, and it can actually be quite a (...) damaging response for the confidence of that person who’s raising it…. So that’s gonna knock their confidence…To do that again.” (P7, safety & governance)*

**5.2. Sub theme:** **Over-reliance on specialists:** Some participants noticed an over-reliance on the diabetes team which led to a decreasing expertise in other staff groups and fostered a perception that diabetes care should only be done by the professionals perceived to be responsible for it. This led to delays when the team was not available, which then compromised safety:

*“And I think generally speaking, over the last five years or so because people now have just relied so heavily on the diabetes team (...) we’ve just, we now (…) probably wouldn’t even know what to turn around and put them on to.” (P17; pharmacist)*

#### Theme 6: System learning following insulin related incidents.

Most participants felt it was important to be proactive and review incidents appropriately to identify and implement methods of improving insulin safety.

**6.1. Sub-theme:** **Absence of systems approach:** Many participants felt a system-based approach to safety was not embedded within their organisation. Instead, the service appeared to have a “*culture of being reactive to harm*” *(P14; nurse)*. Participants were therefore frustrated by witnessing missed opportunities to prevent harm:

*“(...) You can almost see things becoming a trend or becoming a pattern and you’re escalating it, but it it’s not until it gets to a point where someone’s really ill or… a significant, what I would see as a significant harm happens that you sometimes get a response (...).” (P1, nurse)*

Barriers to reporting incidents, included lacking time to submit reports and confusion about completing patient safety and governance questions. Staff also feared repercussions, feeling that reporting mistakes tended to elicit a punitive “*you have done something wrong, you have made a mistake*” *(P14; nurse)* approach from the service. Safety reviews were referred to as archaic and limited to personal reflections by a single member of staff with no wider systemic factors considered. Several staff members felt strong leadership was required to affect any cultural shift in the service:

*“…I think the senior management also have to like, learn that they have to like put their initial impression on the side when they are kind of investigating these things….and that should not impact that person, future experiences and interactions with you.” (P8; doctor)*

**6.2. Sub-theme:** **Learning from what goes well:** Staff recognised the need for more system sharing of learning from incidents to enable systemic improvements in insulin safety:

“*... Need to... Do a little bit more of system sharing and learning,(...) We need to empower people to think how... (...) where do I go with it and who do I share it with? (...)” (P5; safety & governance)”*

**Fig 2:**
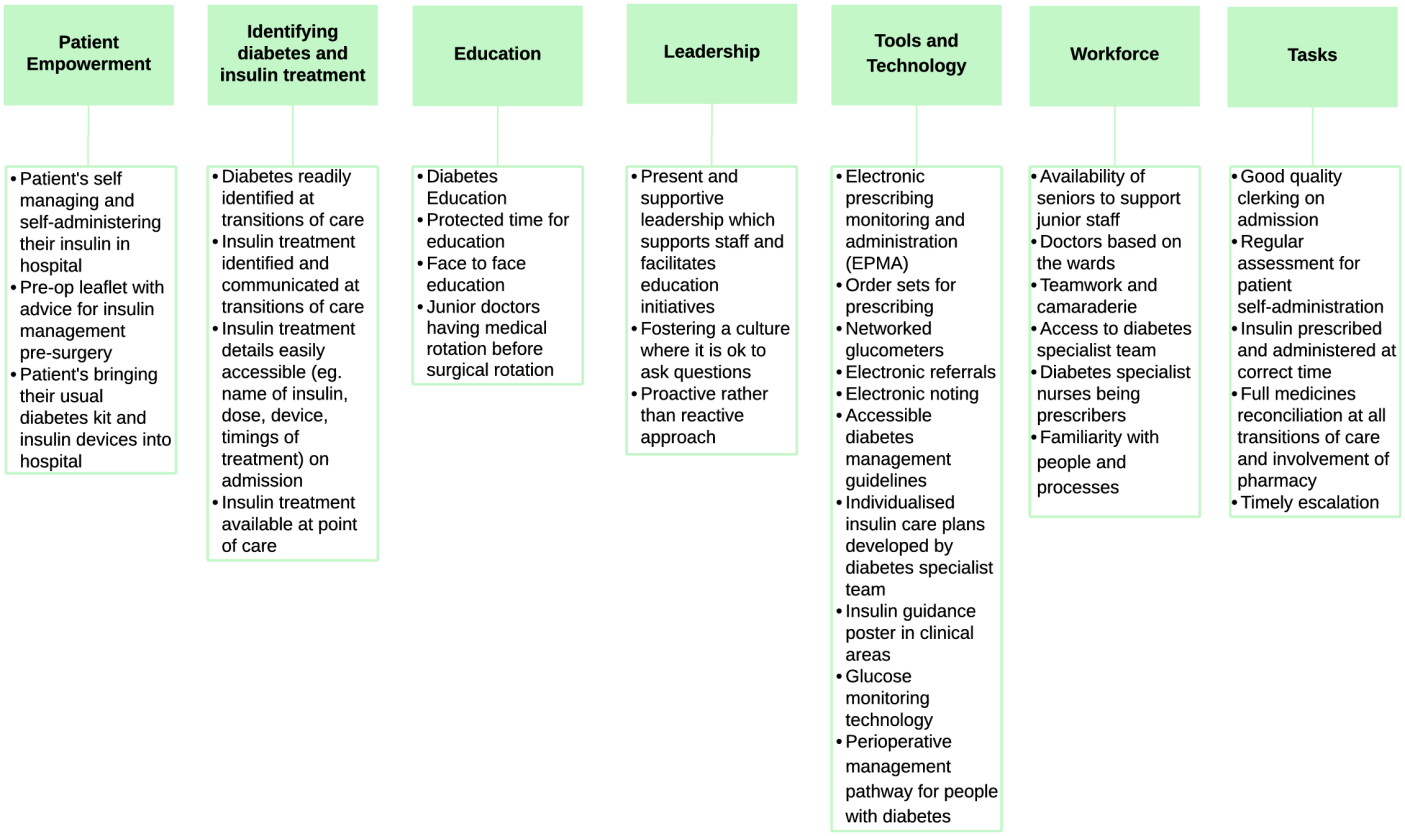
NHS staff perspectives on “Learning from what we do well or what we find helps insulin safety”.

Staff emphasized the importance of disseminating safety information more widely for those who cannot attend safety reviews. There was also a reliance on digital communication, which participants acknowledged may not be as accessible to busy frontline workers. Staff wanted to move away from negativity and towards adopting appropriate acknowledgement and recognition for staff. Professionals were able to identify different factors which enhanced insulin safety, which were related to patient empowerment, identifying diabetes and insulin management, education, leadership, tools and technology, workforce, tasks, and are summarized in Fig 2.

## 4. Discussion

This study has highlighted the complexities of insulin management in practice as experienced by hospital staff. Participants revealed an array of problems with a disjointed system and identified patient and contextual elements impacting on inpatient insulin safety.

Despite having a perioperative pathway guideline at the study site, there was still a lack of patient-centred joined up working and several gaps between recommendations/work as imagined, and care delivered/work as done. Implementation gaps in perioperative inpatient diabetes care recommendations have been recognised previously [19,22,23]. Initiatives such as the Ipswich Perioperative Pathway of Patients with Diabetes (IP3D), can improve outcomes and efficiency [22]. A peri-operative care passport for people with diabetes and support from a perioperative diabetes specialist nurse were among key components. Whilst the importance of diabetes specialist staffing can provide a high return on investment there is still significant variability in hospitals across the UK, and obtaining permanent funding for the resources to implement such pathways can be challenging [38]. Our participants also spoke about the difficulty of implementing guidelines, for example, with regards to perioperative use of intravenous insulin infusions. Indeed, the impact on safety of misalignments and adaptations in the management of IV insulin in practice has been reported previously, across international literature [11,39,40]. Clearly a better understanding of what actually happens in practice is needed to develop more resilient systems of care which support flexible standards and safe adaptations in response to unpredictable events.

Our sample highlighted the need for more standardized training on diabetes and insulin management. Internationally, knowledge and confidence gaps in insulin knowledge amongst the workforce have previously been identified [5,23,25,40]. In our study, there was a fear of causing hypoglycaemia reported among staff, recognising the increased risks associated with hypoglycaemia in the older or more frail population. Increased risks of hypoglycaemia are recognised for older adults, and often hypoglycaemia avoidance is a goal of care in this population [41]. The hospital environment has many factors that increase hypoglycaemia risks [31]. This coupled with additional factors which may be more prevalent in the older population such as physiological changes, presence of multiple healthcare conditions, frailty, polypharmacy, nutritional, communication deficits, reliance on others for care [41] increase the challenge to safely manage insulin therapy in this population. Balancing hypoglycaemia avoidance whilst maintaining target glucose post-operatively and avoiding symptomatic hyperglycaemia is complex [2].

Previous research suggests anxiety associated with causing hypoglycaemia may prevent nurses from adhering to insulin management protocols [42] and that consequences of hypoglycaemia are perceived as more significant than those of hyperglycaemia [23] However, staff turnover in hospitals as well as the rapid pace of developments in diabetes and insulin management, make it a challenge to deliver continuing education on insulin safety [25]. Equally, there is wider concern about the lack of multi-professional undergraduate preparation for diabetes inpatient care [43,44]. Clearly, pre and post qualification training for all professionals needs to target the complex system, cultural and relational aspects implicated in hospital insulin management. Training also must acknowledge the additional considerations of managing insulin safely in the older population, especially given the rising prevalence of older adults with type 1 diabetes [41]. This will empower staff to apply knowledge in practice, make safe adaptations to care, manage unpredictability and have the confidence to challenge suboptimal or outdated practices. Simply relying on top-down approaches to improve safety will not work in complex adaptive systems [45]. Novel educational and reflective approaches aimed at empowering training and junior prescribers to deal with ambiguity and complexity of insulin prescribing and change culture and practices for safer and enhanced experiences of care have been reported [44].

Staff in this study reported how difficult it can be to access accurate insulin dosing information from older people who may be clinically unwell or frail on admission, and reported concerns about quality of clerking and limitations of using electronic records for dosage information. There may be an opportunity to consider how preparedness for hospital admission can be enhanced amongst the older population/their carers to improve hospital insulin safety; noting that staff in this study reported patients did not always bring their insulin to hospital, assuming the hospital would have it.

A recent audit study in a UK hospital highlighted how insulin prescribing errors on admission occur frequently and this was often linked to the source of information for insulin management which was used on admission and thus warrants review [46]. Transition between services also presents a high risk for medication errors in other studies [40,47]. For example, ineffective communication at surgical transitions of care (e.g., between ward and theatre) has been implicated in insulin errors which have resulted in intraoperative hypoglycaemia [40].

Several participants acknowledged that, although rarely reported, insulin timing administration errors were frequent in practice; nurses reported difficulties with administering insulin on time. Mismatches in the coordination of administration of meal delivery, glucose monitoring and bolus insulin administration and delayed insulin administration have been identified in other studies [24,48,49]. Whilst US studies attempting to improve timing of insulin administration in hospital have shown this is a complex problem, there are efficiency and cost savings and improved patient safety when system approaches are implemented [24,50]. Further efforts to understand and improve nursing and hospital practices with regards to timely administration of insulin are needed.

Promoting safe self-administration of insulin can play a key role in improving timing and safety of hospital insulin administration, reduce harm and improve patient satisfaction and experience [51]. However, data from the latest UK National Diabetes Inpatient Safety Audit (NDISA) shows only 63.4% (90/142) hospitals surveyed had such a policy for promoting self-administration in place [52]. In this study site, despite having a self-administration policy, it was not always implemented effectively. Indeed, the older age of patients in particular, appeared to decrease staff motivation for encouraging self-management. Further research is therefore needed to develop and maintain the safe effective implementation of self-management initiatives, particularly in older or more frail populations.

Staff in our study also spoke about dispersed ownership of diabetes care and responsibility across care pathways, which hindered coordinated and timely diabetes care as well as preventing system improvements. Similar issues have been reported in a qualitative study by Rousseau et al., [25] who suggested safe insulin therapy requires interventions by several different professionals, but that these staff may not have or perceive they have overall responsibility for glycaemic management. This study also highlighted the importance of existing relational infrastructure, politics, power dynamics and culture on insulin safety. The role hospital culture plays in patient safety has been recognised in previous research [53] Indeed, the importance of strong leadership, continuous learning and improvement and a compassionate culture where staff can speak freely is key to NHS patient safety strategy [52]. This study and others, show how in practice, these aspects are challenging to change and embed; yet are an essential element for multi-component interventions targeting patient safety [39].

From our interviews, it appears many insulin errors may remain unreported. This variability in reporting and underreporting diabetes related harms has been previously identified as an issue in the UK [43,52]. However, this study found the way insulin incidents and safety were often explored in hospital did not always consider the multiple interacting components in insulin use, instead focusing on the behaviour of one or two individuals. Multiple methods are required to obtain patient and staff feedback on actual insulin errors and to drive improvements to strengthen system resilience. Equally it is important to share learning and good practice when insulin management is done well within this complex adaptive system [39,45]. Staff interviewed in this study felt this was not done enough in their service, suggesting an opportunity to engage and involve staff in enhancing hospital insulin safety.

### 4.1. Study strengths and limitations

This is the first known qualitative paper to capture day-to-day reported experiences and perspectives of multiprofessional staff caring for older adults with diabetes across the surgical pathway. However, data collection was based on a single UK site, and thus our findings may not be transferable to healthcare professionals working in other UK hospitals or indeed internationally. The nursing profession made up over 50% of the sample and so their experiences of insulin use may be over-represented in the data. In addition, staff who did not want to participate in the study may have different views to those who were interviewed. Finally, the lead researcher (CLF) worked clinically at the research site and was known to some, but not all of participants recruited. However, reflexivity techniques and a team approach were employed, with data analysis interpretations regularly discussed with the other co-authors to attenuate any bias.

## 5. Conclusions

This study provides staff insights into some of the multiple interacting components which influence insulin safety in the care of older or frail adults undergoing surgical hospital admission. Some of these factors may be applicable to wider hospital settings.

The use of research methodologies and engagement with a diverse range of stakeholders will help better understand care misalignments, unpredictability and staff adaptations in practice. This will support the design, implementation and evaluation of flexible interventions which can increase hospital insulin safety.

## Supporting information

S1 File Standards for Reporting Qualitative Research (SRQR) checklist. (PDF)

S2 File Research site characteristics. (PDF)

S3 File Sample interview questions. (PDF)

S4 File Preliminary codes, initial thematic framework and final themes and subthemes. (PDF)

S5 File Additional representative quotes. (PDF)

S6 File Graphical Abstract. (PDF)
