# Supplementary material for: “It is a false safety net”: A qualitative exploration of multiprofessional staff experiences of insulin management in hospitalised older or frail adults with diabetes undergoing surgery
Source: PLoS One. 2025 Oct 7;20(10):e0332088. doi: 10.1371/journal.pone.0332088 (PMC12503304; doi:10.1371/journal.pone.0332088)
Supplement: S2 File — (PDF) [file pone.0332088.s002.pdf]

S2 File. Research site characteristics

RESEARCH SITE CHARACTERISTICS	
**HOSPITAL CHARACTERISTICS**	District General Hospital in rural area of England. Serving a population of 235000 people, in an area with higher than national prevalence of older adults with diabetes.<br>The only secondary care hospital in the county, which also serves neighbouring county, in Wales.<br>Part of a Foundation Group of 4 hospitals across different counties.<br>The research site has not yet undergone Diabetes Care Accreditation Programme (DCAP). DCAP
**DIABETES INPATIENT TEAM**	Consultant led diabetes specialist inpatient team with diabetes inpatient specialist nurses (DISN) works Monday to Friday 9AM-5PM.<br>There is a multidisciplinary foot team for diabetes. There is no dedicated inpatient diabetes specialist pharmacist, dietitian or psychologist in post.
**PERIOPERATIVE CARE**	There is a peri-operative pathway based on Centre for Peri-Operative Diabetes Guideline.<br>There is no Perioperative DSN.
**DIABETES AND INSULIN SAFETY**	Diabetes inpatient team meets weekly to discuss incidents and complaints.<br>There are quarterly diabetes safety forum meetings in place with patient safety, organizational leads, IT and analytic teams invited where thematic review of incidents and harms are discussed.
**GUIDELINES RECOMMENDED BY JOINT BRITISH DIABETES SOCIETIES**	The hospital uses guidelines for diabetes hospital care based on the JBDS-IP guidelines, available on the Trust intranet.<br>There is a guideline in place for emergency alternatives to unavailable insulin preparations.
**INPATIENT SELF-MANAGEMENT OF DIABETES AND INSULIN**	There is a self-management policy for diabetes.
**TRAINING**	Online safe use of insulin e-learning module is mandatory for relevant clinical staff.<br>Diabetes and insulin safety included in junior doctors induction and training.
**ACCESS TO CARBOHYDRATE CONTENT OF MEALS**	Carbohydrate content of hospital meals is available on request.

**IDENTIFICATION OF DIABETES ON ADMISSION AND REFERRAL TO INPATIENT DIABETES TEAM.**	There was no electronic system to identify all people with diabetes on admission at the time of the study. EMIS view is integrated with the electronic patient record (EPR) allowing access to GP records, however only available for patients residing in the county.<br>There is an electronic pathway to refer inpatients with diabetes for inpatient diabetes specialist review.
**ELECTRONIC PRESCRIBING SYSTEMS AND SUPPORT**	Electronic prescribing, monitoring and administration (ePMA) is in place with several inbuilt insulin related order sets developed for sub-cutaneous and intravenous (IV) insulin infusion prescribing.<br>Hospital guidelines guide insulin rate prescribing during IV insulin Infusion use and management.
**INSULIN ADMINISTRATION**	IV insulin infusions are prepared and set up by nurses in clinical areas guided by hospital protocols. IV insulin monitoring and management is recorded on paper charts at the patient bedside. Bar coded medicines administration is not used. Two nurses conduct independent verification of insulin prescription and device prior to insulin administration.
**WEB-LINKED BLOOD GLUCOSE AND KETONE METERS**	Point of care monitoring of capillary blood glucose (CBG) and ketones is undertaken via networked web-linked meters; however, these do not directly upload patient result into EPR. Web-linked networked glucose and ketone levels are monitored by inpatient diabetes specialist nursing team to prioritise in-reach inpatient reviews.
**DISCHARGE CHECKLIST**	The hospital has electronic discharge system but there is no specific diabetes discharge checklist in place.
**DIABETES INPATIENT HARMS AND PARTICIPATION IN AUDITS**	The hospital participates in National Diabetes Inpatient Safety Audit (NDISA) reporting patient harms related to hospital acquired diabetic ketoacidosis, hyperosmolar hyperglycaemic state, severe hypoglycaemia and foot lesions/ulcers.<br>National Diabetes Inpatient Safety Audit - NHS England Digital<br>The hospital submits data to DEKODE-DKA quality improvement project.<br>DEKODE—A cloud-based performance feedback model improved DKA care across multiple hospitals in the UK
