# Supplementary material for: “It is a false safety net”: A qualitative exploration of multiprofessional staff experiences of insulin management in hospitalised older or frail adults with diabetes undergoing surgery
Source: PLoS One. 2025 Oct 7;20(10):e0332088. doi: 10.1371/journal.pone.0332088 (PMC12503304; doi:10.1371/journal.pone.0332088)
Supplement: S3 File — (PDF) [file pone.0332088.s003.pdf]

Examples of semi-structured interview questions with staff:

Main questions

- Could you tell me about your role/tasks in the context of insulin use in hospital?
...And specifically regarding older/frail adults with diabetes admitted on [names of wards]
- What do you think is important in relation to insulin safety in hospital?
- Which types of insulin errors have you come across in your professional experience?
- Have any of your patients or patients in the area you are working and who are treated with insulin experienced hypoglycaemia (low glucose levels)?
- Have any of your patients or patients in the area you are working and who are treated with insulin experienced hyperglycaemia (high glucose levels)?
- Could you tell me how insulin errors are currently reported?
- Could you tell me how insulin errors are currently investigated?
- How do you think we could improved different areas of the reporting process?
- How do you think we could improve different areas of the insulin review process?
- What opportunities are there for patients/their carers to be involved in their insulin management in hospital?
- We know many factors influence insulin safety in hospital: patient factors, healthcare professional factors and context/system factors. What do you think would improve insulin safety and reduce insulin errors?
- What sorts of materials or resources might be helpful in improving insulin safety and reducing insulin errors?

Probing themes:

Teamwork, communication, time critical coordination, 6 Rights of insulin safety, Accessibility of information, Perioperative care pathway and care transisions: pre-op, intra-operative care and surgery, early post-operative phase, post acute care; Higher risk situations, Personal, team, area/ward, system levels, learning and change; patient factors, HCP factors, context, system factors, Empowerment

Examples of probing questions

- Could you tell me a bit more about... that?
- Could you tell me step by step what that involves?
- What were some memorable experiences (good or bad)?
- What do you find challenging in ...?
- How did you feel about that?
- How was it dealt with?
- What were the things that were said and done afterwards?
- Can you give me a couple of examples?
- Can you give me some examples of when this worked well and why? ...not worked well and why?
- Which other professionals do you see as part of the team involved in insulin management? How do you communicate with them? How do you access support... from who? How do you provide support?
- Can you identify why ...? Can you identify factors such as time of day/night, out of hours, weekends, certain point in the perioperative pathway...?
- Could you tell me your overall impression of how that works?
- Do you think there is learning from insulin error incidents? Does that learning lead to change? Can you give some examples?
- Do you think self-management of insulin is supported and facilitated for this cohort of patients? What might stop it from happening?
- Do you think self-management in hospital is important to the patient?
- If the patient has family members or carers are these involved in any way during their hospital admission and discharge?
- In what circumstances would this/these help?
