# Supplementary material for: “It is a false safety net”: A qualitative exploration of multiprofessional staff experiences of insulin management in hospitalised older or frail adults with diabetes undergoing surgery
Source: PLoS One. 2025 Oct 7;20(10):e0332088. doi: 10.1371/journal.pone.0332088 (PMC12503304; doi:10.1371/journal.pone.0332088)
Supplement: S4 File — (PDF) [file pone.0332088.s004.pdf]

Preliminary Codes

311 Initial Codes

Examples: Accessibility of information; Dose of insulin unknown; Finding out insulin dose; Adherence to guidelines; administration of insulin; admission to ward; Anxiety; Assumptions; Availability of seniors; Barriers to reporting; Blame; Boarding patients; Care of older and frail adults; catering; Changing plans; Chaotic times; Check and challenge; Check glucose levels; Communication; Culture around insulin; Delay in insulin review post hypo; Delirium post surgery; Diabetes Team; Diet in hospital;Digital communication; Discharge planning; Dispersed responsibility; Documentation; Drug round; Education; Elderly; Electronic documentation; Electronic prescribing; Emotional toll; Empowering staff; Enablers to go well; Engaging staff; Expectations; Experience; External environment factors; Factors affecting timing of insulin; Fear; Feel bad; Forget; frustrating; functional loss; Gap between WAI and WAD; Handover of information; Handover sheet;Hospital Spaces; Locations within hospital; Human error; Identifying diabetes; Ilness; Impressions count; Inbuilt alerts; Incident reporting as a challenge; Incident review; Individualised care; Induction; injection technique; Insulin not available; insulin passport;Intravenous insulin; involving the patient; knowledge gap; Locked away; Mandatory training; medication storage; Misalignment; mismatch glucose, food, insulin; Misplaced; Multiple related tasks; Need for empathy; ; Never ending; Nil by mouth; no prompt; No time factor; Not checking; Not knowing what to do; Out of area patients; Out of hours; Patient choice; Patient cognitive status;Patient disempowerment; Patient flow; Patient records; Perceived causes of insulin errors; Perioperative pathway; Personal reflection post incident; Power dynamic; Prescribing as a challenge; Reasons for witholding insulin; Review of glucose levels; Review of insulin doses; second check for insulin administration; Self-management in hospital; Sources of support; Staff breaks; Staff Perceived causes of hypoglycaemia; Staffing turnover; Stigma; stressful; Support to staff; Tasks clerking; team huddle; Teamwork; time critical coordination; tired; told off; Training gap; transfer from IV to SC insulin; transitions of care; Trauma patients; Types of insulin errors; Uncertainty; Workload pressures

Initial thematic framework

1. PATIENT FACTORS
1.1 Elective/Emergency
1.1.1 Illness
1.1.2 Trauma
1.2 Patient Choice
1.3 Patient assumptions
1.4 Cognitive function
1.4.1 Post-operative delirium
1.5 Frailty level and function
1.6 Co-morbidities
1.7 Nutritional Status
1.7.1 Fluid Status
1.7.2 Nil by mouth
1.7.3 Dietary intake
1.8 Type of diabetes
1.8.1 Diabetes treatment
1.8.2 Level of self-management
1.9 Support network
1.9.1 Family/carers
1.9.2 District nurses

2. PATIENT EMPOWERMENT ETHOS
2.1 Assumptions about elderly
2.2 Practical considerations
2.2.1 Assessing and facilitating self-administration
2.2.2 Access to insulin
2.3 Previous experiences
2.3.1 Positive
2.3.2 Negative
2.4 Risk Aversion
2.5 Perceptions
2.6 Control

3. EXTERNAL ENVIRONMENT
3.1 Socio-economic factors
3.2 Insulins available
3.3 Supply
3.4 Universities

4. TRANSITIONS OF CARE
4.1 Places of Care
4.1.1 Pre Op
4.1.2 ED
4.1.3 Ward
4.1.4 Theatre
4.1.5 Recovery
4.1.6 ITU
4.1.7 Discharge destination
4.2 When
4.2.1 Out of hours
4.2.2 In hours
4.2.3 Day or night
4.2.4 Pre-Op or Post Op
4.3 Next shift/team
4.3.1 Handover of information

5. SYSTEM LEARNING AS A CULTURAL CHALLENGE
5.1 Barriers to reporting
5.1.1 Focus on outcome of lapse
5.1.2 Time
5.1.3 Culture
5.1.4 Fear of repercussions
5.1.5 Impressions count
5.1.6 Unrecognised lapses
5.2 Incident review process
5.2.1 Personal reflection
5.2.2 Leadership to model the cultural change
5.2.3 Not enough systems exploration
5.2.4 Learning for improvement
5.2.5 Approach seen as punitive
5.3 System learning as a challenge
5.3.1 Dissemination of learning
5.3.2 Focus on training
5.3.3 Focus on individual
5.3.4 Learning from what we do well
5.3.5 System changes
5.3.6 Reactive vs proactive

6. MULTIPLE TASKS NOT ALWAYS SEEN AS CONNECTED
6.1 Clerking on admission
6.1.1 Identifiying diabetes
6.1.2 Identifying insulin treatment
6.2 Insulin use process tasks
6.2.1 Prescribing
6.2.2 Administration
6.2.3 Dispensing
6.2.4 Medicines reconciliation
6.2.5 Storage
6.2.6 Transcribing
6.2.7 IV insulin use and management
6.3 Checking and reviewing glucose levels
6.4 Discharge planning

7. TOOLS AND EQUIPMENT
7.1 Ease of use and accessibility
7.2 Integration
7.3 Handover sheet
7.4 Guidelines and protocols
7.5 Insulin and diabetes kit
7.5.1 Supply and Access
7.5.2 Availability
7.6 Patient record
7.6.1 Accessibility of information
7.6.2 Electronic prescribing

8. INTERNAL ENVIRONMENT AND ROUTINES
8.1 Embedded practices
8.2 Mismatch glucose check, insulin and food
8.3 Drug round
8.4 Ward round
8.5 Housekeeping
8.6 Physical layout of place of care
8.7 Busyness
8.8 Staff breaks

9. MULTIPLE STAFF ROLES AND DISPERSED RESPONSIBILITY
9.1 Confidence
9.2 Experience
9.3 Roles and Responsibilities
9.4 Escalation chain
9.5 Competing demands
9.6 Knowledge
9.7 Attitudes and behaviours
9.8 Priorities

10. TEAMWORK AND COMMUNICATION
10.1 Digital communication
10.2 Verbal communication
10.3 Documentation

11. EMOTIONAL IMPACT
11.1 Positive emotions
11.2 Negative emotions
11.3 Need for empathy
11.4 Coping

12. SOCIO-RELATIONAL INFRASTRUCTURE
12.1 Sources of support
12.2 Expectations
12.3 Assumptions
12.4 Agency
12.5 Hierarchical bias
12.5.1 Power dynamics
12.5.2 Impressions count
12.5.3 Control

13. ORGANIZATIONAL FACTORS
13.1 Governance
13.2 Staffing
13.2.1 Nurse to patient ratio
13.2.2 Diabetes team
13.2.3 Staffing turnover
13.2.4 Availability of seniors
13.3 Organizational pressures
13.3.1 Patient flow
13.3.2 Boarding patients
13.3.3 Theatre lists
13.4 Escalation and staff empowerment
13.4.1 Options available
13.4.2 Releasing staff
13.5 Culture

1, 2 & 12 →
1, 3, 4, 8, 9 & 13 →
6, 9, 10 & 12 →
10, 11 & 12 →
5 →
1, 2, 3, 4, 5, 6, 7, 8, 9. 10 & 13 →

Refined framework categories and subcategories

1. PATIENT EMPOWERMENT
1.1 Patient factors
1.2 Power dynamic HCP-patient Healthcare professionals attitudes beliefs and experiences
1.2.1 Assumptions
1.2.2 Recognising value of self-administration
1.2.3 Giving control back to the patient
1.2.4 Navigating cultural differences, inherent biases; approaches to care
1.3 Organizational factors, processes and equipment at point of care
1.3.1 Processes, tools and equipment hindering the self-administration process
1.3.2 Resources, processes, tools and equipment facilitating the self-administration process
1.3.3 Navigating operational pressures and staffing challenges which impact on care and patient disempowerment
1.3.4 General hospital admission factors
Other

2. NON-EQUILIBRIUM, UNCERTAINTY AND AMBIGUITY
2.1 Coping with constant change
2.2 External factors
2.3 Managing changing circumstances, uncertainty and ambiguity in decision making
Other

3. CAPABILITY, CONFIDENCE AND DISPERSED RESPONSIBILITY
3.1 Attitudes, behaviours, beliefs, values and experiences
3.2 Staff knowledge gaps
3.3 Feeling disempowered
3.4. Wanting more knowledge and training
3.5. Capability at point of care
3.6 Accessing support
3.7 Organization and operational factors
3.8 Teamwork
3.9 Feeling overwhelmed or afraid of insulin
3.10 Power dynamic HCP-HCP
3.10.1 Confidence to check and challenge
Other

4. EMOTIONAL AND SOCIO-RELATIONAL ASPECTS
4.1 Communication
4.1.1 Style
4.1.2 Method
4.2 Power dynamics
4.3. Sources of support
4.4 Impressions count
4.5 Emotions
4.5.1 Positive emotions
4.5.2 Negative emotions
Other

5. SYSTEM LEARNING AS A CULTURAL CHALLENGE
5.1 System learning
5.1.1 Dissemination of learning
5.1.2 Learning from what we do well and what helps
5.1.3 Moving towards proactive system changes
5.1.4 Suggestions for improvement
5.1.5 Types of insulin error
5.2 Incident or safety reporting and review process
5.2.1 Personal reflection
5.2.2 Reporting incidents
5.2.3 Undertaking a review
5.2.4 Leadership to model cultural change
5.2.5 Patient involvement
5.2.6 Not enough systems exploration
5.2.7 Learning for improvement
5.2.8 Approach seen as punitive
5.3 Barriers to reporting incidents
5.3.1 Unrecognised lapse
5.3.2 Focus on outcome
5.3.3 Process of reporting
5.3.4 Time
5.3.5 Culture
5.3.6 Fear of repercussions
5.3.7 Impressions count
Other

6. MISALIGNMENTS AND TIME CRITICAL COORDINATION
6.1 Waiting for decision making
6.2 Communication
6.3 Access to insulin
6.4 Accessibility of information, usability of systems and their integration
6.5 Organization and operational factors
6.6 Navigating transitions of care and access to the right information
6.7 Patient Factors
6.8 Identifying diabetes
6.9 Staff and Staffing factors
6.10 Physical layout or spaces of care
6.11 Tools and equipment
6.12 Escalation and referral
6.13 Insulin use and management process tasks
6.14 Insulin use and management amongst other tasks of care and routines
6.15 Mealtimes & Food
Other

6 →
2 →
3 →
1 →
4 →
5 →

Final Themes and subthemes

Transitioning through care: access to relevant information to diabetes and insulin
- Access right information at the right time
- Dispersed staff responsibility

Coping with changing circumstances and ambiguity
- Operational Complexity
- Organizational pressure

Staff knowledge and confidence gaps; wanting more training
- Feeling anxiety of insulin management
- Staff knowledge and gaps

Fostering patient empowerment
- Inflexible hospital procedures
- Staff attitudes towards self-administration

Staff support systems
- Value of support
- Over-reliance on specialists

System learning following insulin related incidents
- Absence of systems approach
- Learning from what goes well
