# Supplementary material for: “It is a false safety net”: A qualitative exploration of multiprofessional staff experiences of insulin management in hospitalised older or frail adults with diabetes undergoing surgery
Source: PLoS One. 2025 Oct 7;20(10):e0332088. doi: 10.1371/journal.pone.0332088 (PMC12503304; doi:10.1371/journal.pone.0332088)
Supplement: S5 File — (PDF) [file pone.0332088.s005.pdf]

Supplemental File: Additional representative quotes

“It is a false safety net”: A qualitative exploration of multiprofessional staff experiences of insulin management in hospitalised older or frail adults with diabetes undergoing surgery.

Learning from what works well and what helps insulin safety: perspectives from NHS staff

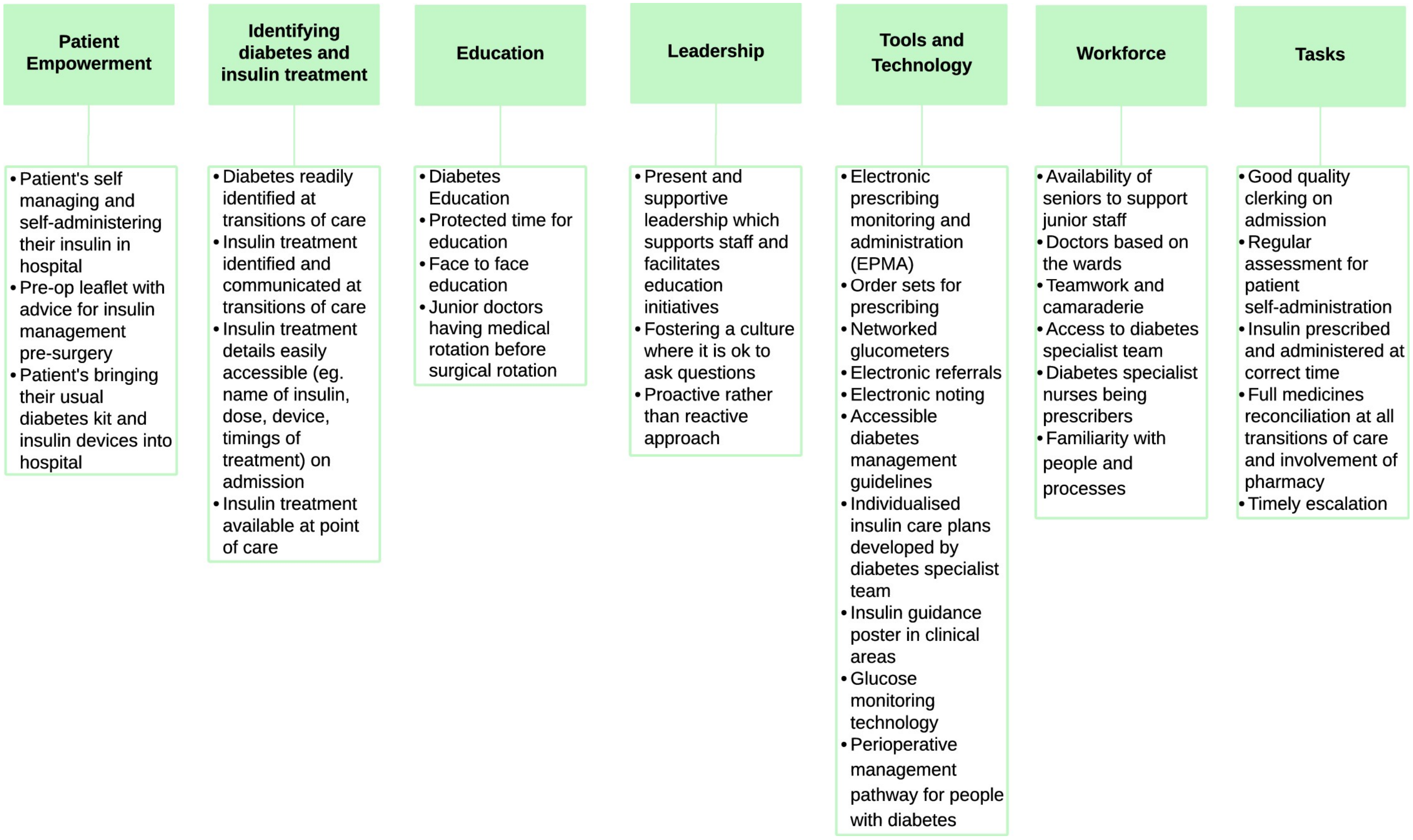

**Representative quotes:** Learning from what works well and what helps insulin safety: perspectives from NHS staff	
Patient Empowerment	• *“Because self administration at the end of the day makes their lives so much easier anyway. They don't have to get a double signature in the middle of a busy drug ground, which can take forever, that patients get their insulin on time at the right time. (…)” (P06, nurse)*<br>• *" So if they're coming in with a pilonidal abscess, for example, and then we, if we're lucky enough to be able to get to see them the day before, before they go home to come back in again, we can give them our usual leaflet and we tell them to follow things exactly as we would do for elective surgery." (P21, doctor)*
Identifying diabetes and insulin treatment	• *“I suppose it's just, certainly from a prescribers point of view, when you're clerking somebody, it's doing a thorough history, isn't it? It's doing, it's doing your clerking properly…” (P3, nurse)*<br>• *“Identifying it, so if someone's on insulin, particularly if they are tube fed erm, or about to be tube fed, then identifying it and if I'm honest, I just call the diabetes nurses and let them do the rest of it." (P12, allied health professional)*<br>• *"I think it's really important, obviously, to make sure we've got their doses right and the timings of when they're taking their insulin. (…) it's just making sure we've got it prescribed correctly for them and they're having it (…).At the times that suit them better and you know with, you know, when they're eating or if they're having it at night time or morning. Yeah, because that could be changed." (P9, pharmacy)*
Education	• *“I think the junior doctors who already had their like medical rotations and then they go to the surgical rotations, they are more, they are more confident about the insulin use and prescription specifically. So I think that is a big variable. So that do affect the patient's management.” (P8; doctor)*<br>• *"So we did have that Peri-operative, diabetes nursing management video. That was really helpful for me. And I would have to say. That the class that we had when at the start, because that was a bit more comprehensive." (P20, nurse)*<br>• *"I think [face to face education] I think it would be most people's preference really, especially if you can actually see the insulins and, you know, go through them properly and" (P9, pharmacy)*
Leadership	• *“[ward sister] essentially gathered 2 to 3 nurses each time, said I'll look after your patients and your call bells. You go and do that learning now in my office, away from the ward (..) they couldn't see call bells. They didn't have patients looking for them. They didn't have relatives looking for them. We just shut the door and closed us ourselves away. Yeah that was really good." (P6, nurse)*<br>• *“There are pockets where things work well and that generally is, you can see really engaged and present leadership in those areas. I think if we've got that right, if (…)people are able to get out from behind the computer and go in, you know, sort of show staff by doing and by supporting them, erm, and patients, of course, you know. That really makes the difference " (P14, nurse)*<br>• *“I think the most important thing is that you have to give the confidence to the junior doctors that they can ask questions” (P8, doctor)*
Tools & Technology	• *" I think there have (…) been improvements since we've had electronic prescribing because we have the order sets to try and prompt people to prescribe things at appropriate times. We get clearer documentation on insulin doses from nursing staff.” (P1, nurse)*<br>• *"(…) it's helped with EPMA because (…) it's harder to make an error (…) because if you say try and (…) prescribe Tresiba twice a day, it will come up with a warning and you've got to acknowledge it. So it makes you, it makes you think.” (P3, nurse)*<br>• *"Here there is a good thing to prescribe, insulin order sets OK, so it so it's easier to prescribe.” (P15, doctor)*<br>• *“Obviously I tried to get familiar with the Intranet guidelines and prescribe it then according to the Intranet guidelines and you can never go wrong with those.” (P11, doctor)*

	●*"there are a few patients who are on (…), insulin care plans. So in those patients, I think things are more easily managed because they have like a specific doses and everything written on that. So it make people easy to I kind of interpret that what to give, when to give (…). So that is one kind of documentation that reflects that if you have more information in hand then you are you can avoid the mistakes and errors." (P8, doctor)*<br>● *"we're a big fan of like the diabetic team coming and giving a proper like individualised regimen. So we really like when it's clear and it's like if the patient is eating well, if they're not eating well, they're really clear and obvious to follow. " (P13, nurse)*<br>● *""we have the poster in our clean utility with all the different types of insulin on and whether to administer that or not. And that is one of the most important things on the ward for me because I always go back to that, if I'm querying it. So I do find that very helpful." (P16,nurse)*<br>●*"And on the new machines, I think that the diabetic team can also look at it remotely as well, can't they? (…) that's really useful because if you have got somebody that you're not sure about or you're thinking well, should I reduce the long term insulin as as well as the the shorter acting insulin you can just put [an electronic] referral through (…) and you know, keep an eye on that for their response." (P3, nurse)*<br>● *"I think now we've got electronic noting it's a lot easier 'cause, you can read handwriting" (P12; allied health professional)*<br>● *"I love them [flash and CGM]. They're fantastic. (…). I think they're, I think they're brilliant and then you can trend them and it tracks them and some of them we can even decide what they're going to do with their insulin pumps afterwards, which is even better. (…) (…) I mean you can see where they've had problems during the night. It sort of helps you predict as to how things might go and they can often show you trends, (…)." (P21, doctor)*<br>● *"And it's great that we've got the perioperative pathway." (P18, nurse)*
Workforce	● *"I think the involvement of or the supervision of the junior doctors in the surgical ward sometime the (…) registrar and the middle grade, they are very busy with their other things. So if they are not that busy then they are more supportive to the junior doctors. So that sometimes help." (P8, doctor)*<br>● *"there are some situations when the patient is very unwell and would still definitely require senior inputs anyway. So for example, diabetic ketoacidosis steps say a medical emergency that you know you definitely want to escalate to a seniors before you do anything. And I guess senior availability for help?" (P11, doctor)*<br>●*" Out of hours is a challenge [without diabetes team]. Yeah. So what, we encourage the doctors also, if nurses are really are not sure about any point we ask them to please consult with the medical team. They can give us a guidance." (P19;nurse)*<br>●*"Whereas now you tend to have that ward based junior doctor and they really are ward based. They're either here doing ward round or completing jobs on the ward or they're doing not hands on things, but they are in the office so they're on site, which is helpful." (P2, nurse)*<br>●*"I would say it's easier for me. I think I've been at this trust for now seven or eight years, so I've generally have got good contacts. (…) I would say that I know how to turn round and erm highlight a concern from my point of view of diabetes. If I was, if I needed to and if I didn't, I'd know at least somebody that would also know. So I might not ever make a referral request. But I would know the process that needs to be taken in order to kind of put a specialty request in, for example." (P17, pharmacy role)*
Tasks	●*"I think making the right assessment and then if if you feel that patient is delirious, then you need to repeat that assessment after a specific time." (P8, doctor)*

	• *“especially if if the whole process has been undertaken. So they've had the meds rec and they've got had their discharge through pharmacy and it's clinically checked then yeah definitely it's it's it's a very smooth process.” (P9; pharmacy role)*<br>• *"So yeah, so we'll speak to the patient first. We're always trying to speak to the patient first try and get a list off them of their current regular medications. And it's always important that we try and talk to them first, because they don't always take the medications, how they're actually prescribed. So it's always good to find out how they take them and hear it from theirself first of all, and then we use like the GP records to compare that against. Any repeat prescriptions we will use that medication that they come in with as well." (P9; pharmacy role)*<br>• *"I think it's usually reviewed quite promptly. We we're we're quite a proactive ward I think for escalating things, even if it's a little thing, we'll escalate it." (P18, nurse)*
