# Supplementary material for: “It is a false safety net”: A qualitative exploration of multiprofessional staff experiences of insulin management in hospitalised older or frail adults with diabetes undergoing surgery
Source: PLoS One. 2025 Oct 7;20(10):e0332088. doi: 10.1371/journal.pone.0332088 (PMC12503304; doi:10.1371/journal.pone.0332088)
Supplement: S6 File — (PDF) [file pone.0332088.s006.pdf]

"It is a false safety net"

A qualitative exploration of multiprofessional staff experiences of insulin management in hospitalised older or frail adults with diabetes, undergoing surgery.

Method

Semi-structured interviews:

Study participants

20 Female
3 Male

Medics (n=4)
Nursing roles (n=12),
Alied health professionals (n=2)
Pharmacy roles (n=2)
Safety & governance (n=3)

Age 27 to 62 years

Ethnicity
White (n=17)
Asian (n=5)
African (n=1)

Results

Six emerging themes

Capability, confidence and dispersed responsibility

Feeling anxiety regarding insulin management
Staff knowledge and gaps
Wanting more training

System learning as a cultural challenge

Absence of systems approach
Reactive to harm
Learning from what goes well

Fostering patient empowerment

Inflexible hospital procedures & system
Staff attitudes and behaviours towards self-administration

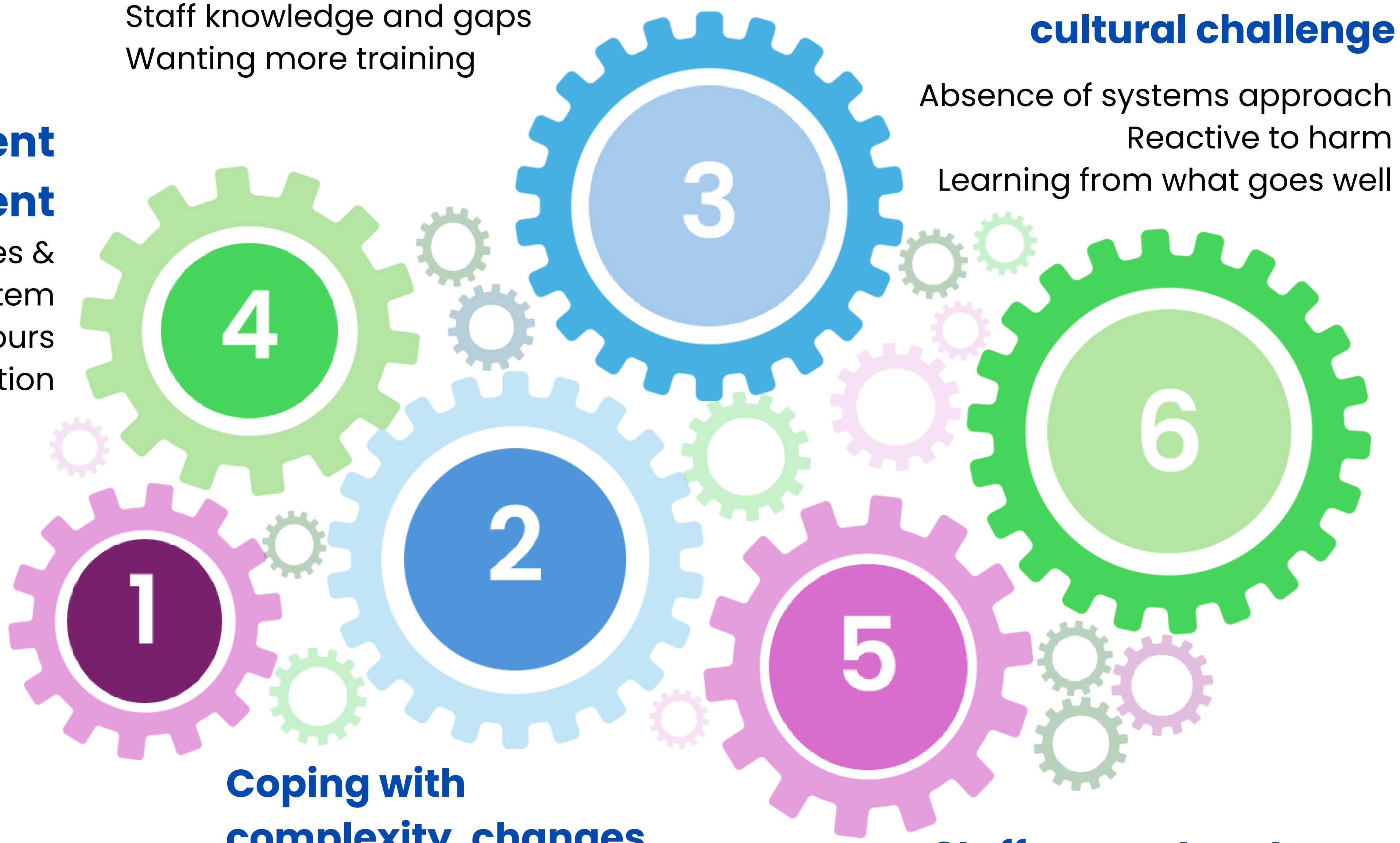

Transitioning through care; interconnectedness and misalignments

Access to the right information, people and equipment at the right time
Dispersed staff responsibility

Coping with complexity, changes and ambiguity

Operational complexity;
Organizational pressure

Staff support systems

Accessing support
Power dynamics
Overreliance on specialists
